# Image Quality Improvement in Adaptive Optics Scanning Laser Ophthalmoscopy Assisted Capillary Visualization Using B-spline-based Elastic Image Registration 

**DOI:** 10.1371/journal.pone.0080106

**Published:** 2013-11-12

**Authors:** Akihito Uji, Sotaro Ooto, Masanori Hangai, Shigeta Arichika, Nagahisa Yoshimura

**Affiliations:** Department of Ophthalmology and Visual Sciences, Kyoto University Graduate School of Medicine, Kyoto, Japan; Glasgow University, United Kingdom

## Abstract

**Purpose:**

To investigate the effect of B-spline-based elastic image registration on adaptive optics scanning laser ophthalmoscopy (AO-SLO)-assisted capillary visualization.

**Methods:**

AO-SLO videos were acquired from parafoveal areas in the eyes of healthy subjects and patients with various diseases. After nonlinear image registration, the image quality of capillary images constructed from AO-SLO videos using motion contrast enhancement was compared before and after B-spline-based elastic (nonlinear) image registration performed using ImageJ. For objective comparison of image quality, contrast-to-noise ratios (CNRS) for vessel images were calculated. For subjective comparison, experienced ophthalmologists ranked images on a 5-point scale.

**Results:**

All AO-SLO videos were successfully stabilized by elastic image registration. CNR was significantly higher in capillary images stabilized by elastic image registration than in those stabilized without registration. The average ratio of CNR in images with elastic image registration to CNR in images without elastic image registration was 2.10 ± 1.73, with no significant difference in the ratio between patients and healthy subjects. Improvement of image quality was also supported by expert comparison.

**Conclusions:**

Use of B-spline-based elastic image registration in AO-SLO-assisted capillary visualization was effective for enhancing image quality both objectively and subjectively.

## Introduction

Imaging technology in optical coherence tomography (OCT) has advanced rapidly and contributed greatly to progress in ophthalmology during this decade[1,2]. Improvement of OCT image quality has been achieved not only by increased retinal scan speed, as represented by the advancement of OCT generations from time-domain OCT to spectral-domain OCT (SD-OCT)[3–7], but also by image processing techniques to reduce speckle noise using multiple-aligned OCT scans[8,9]. Although multiple B-scan averaging can visualize microstructure in OCT more clearly than single B-scans, this technique is based on high-speed SD-OCT scanning, by which B-scans are generated with minimal scanning distortion variation from one scan to another.

 More recently, confocal adaptive optics scanning laser ophthalmoscopy (AO-SLO) has enabled imaging of retinal cells such as photoreceptors and blood cells[10–12]. Adaptive optics (AO) can compensate for aberrations in ocular optics, allowing clear visualization of individual cone photoreceptors in the living eye. As in OCT, multiple scan averaging has been reported as useful in AO-SLO imaging, with averaged AO-SLO images demonstrating photoreceptor microstructure more clearly than single scans[13–15]. However, unlike SD-OCT, averaging of AO-SLO images is challenged by the relatively narrow and magnified scanning area. An interframe position gap produced by eye motion exists between scan frames, and intraframe eye motion produces distortions in frames[16]. Thus, averaging often results in blurry images. To overcome these issues, hardware and software for eye tracking have been developed that can stabilize the frames, such as cross-correlation methods[17] and the KLT (Kanade-Lucas-Tomasi)-SIFT (Scale-invariant feature transform) algorithm[14], and excellent photoreceptor imaging results have been reported using these systems[18].

In addition to photoreceptor observation, AO-SLO video allows noninvasive monitoring of the movement of blood components without contrast dyes[11,19]. Noninvasive visualization of retinal capillaries using stabilized AO-SLO videos was very effective in analyzing the parafoveal capillary network of diabetic patients[20–22]. Because the technique requires no contrast dyes, angiogram can be safely obtained even in patients with minimal changes of that retina, which would be helpful in the early detection of retinal diseases and may also be a powerful tool for researching the pathogenic mechanisms of retinal diseases. Although the effect of video stabilization on vessel visualization was not analyzed in these studies, it was thought to be profound because the capillary image was constructed as a trajectory of small blood cells using multiple frames.

In this study, we applied B-spline-based elastic image registration on AO-SLO videos to interpolate the images for AO-SLO video stabilization using public domain software ImageJ. B-splines have proven very useful in modeling deformations in many biomedical imaging problems and ensure high-quality interpolation[23–27]. The effect of elastic image registration on AO-SLO-assisted capillary visualization in normal eyes and various retinal diseases was then evaluated objectively and subjectively.

## Methods

This study was approved by the Institutional Review Board and the Ethics Committee at Kyoto University Graduate School of Medicine and performed in accordance with the tenets of the Declaration of Helsinki. Written informed consent was obtained from each participant after a detailed explanation of the nature and possible consequences of the study procedures. 

### Subjects

Twenty-four healthy subjects with no history of ocular or systemic diseases (mean ± standard deviation [SD] age, 35.2 ± 8.1 y) and 25 patients (mean age ± SD, 56.7 ± 13.1 y) diagnosed with various eye diseases at Kyoto University Hospital (5 patients each with diabetic retinopathy [DR], idiopathic macular telangiectasia [MacTel], epiretinal membrane [ERM], central serous chorioretinopathy [CSC], and glaucoma) were recruited in this prospective cross-sectional study.

### Adaptive Optics Scanning Laser Ophthalmoscopy Imaging

The AO-SLO system developed by Canon Inc. was employed for this study[28], with 840-nm imaging light wavelength and 32-Hz frame rate. The imaging light exposure level was set to less than the maximum permissible exposure indicated by the American National Standards Institute[29]. The scan area at the retina was 2.8 × 2.8° and sampled at 400 × 400 pixels. AO-SLO videos were acquired randomly from 1 eye of each subject after pupil dilation with one combined application of tropicamide (0.5%) and phenylephrine hydrochloride (0.5%) and recorded for 2 s per scan area; 6–12 scan areas were collected per subject to cover the parafoveal area. AO-SLO imaging was focused on the photoreceptor layer to enable detection of moving bright objects in the capillaries, which may correspond to leukocytes or plasma gaps as described previously[30].

### Video Processing

Prior to linear and nonlinear registrations, all images were desinusoided in preprocessing to cancel the difference in reflective values between the center and near edge of each image caused by sinusoidal movement of the resonant scanner. 

#### Video Stabilization

For each subject, a 2-s video was randomly chosen for analysis. All videos were first stabilized by linear image registration to align frames, followed by nonlinear image registration to correct image distortion produced by intraframe eye motion. The image quality of constructed capillary images was compared before and after nonlinear image registration. All digital images were processed by a single operator (A.U.) using ImageJ (developed by Wayne Rasband, National Institutes of Health, Bethesda, MD; available at http://rsb.info.nih.gov/ij/index.html). Grayscale ranged from 0 (black) to 255 (white).

#### Linear Image Registration

To align position gaps induced by eye motion across sequential frames, the Stackreg plug-in for ImageJ was used[31]. Stackreg is based on an automatic sub-pixel registration algorithm that minimizes the mean square difference of intensities between a reference image and source images. Rigid-body transformation, by which source images are transformed with translation and rotation to match a reference image while maintaining the distance between any pair of landmark points in the images, was applied to all videos. After registration, videos were cropped to eliminate the margin lacking a retinal image, which was a by-product of registration. 

#### Elastic (Nonlinear) Image Registration

**Figure 1:**
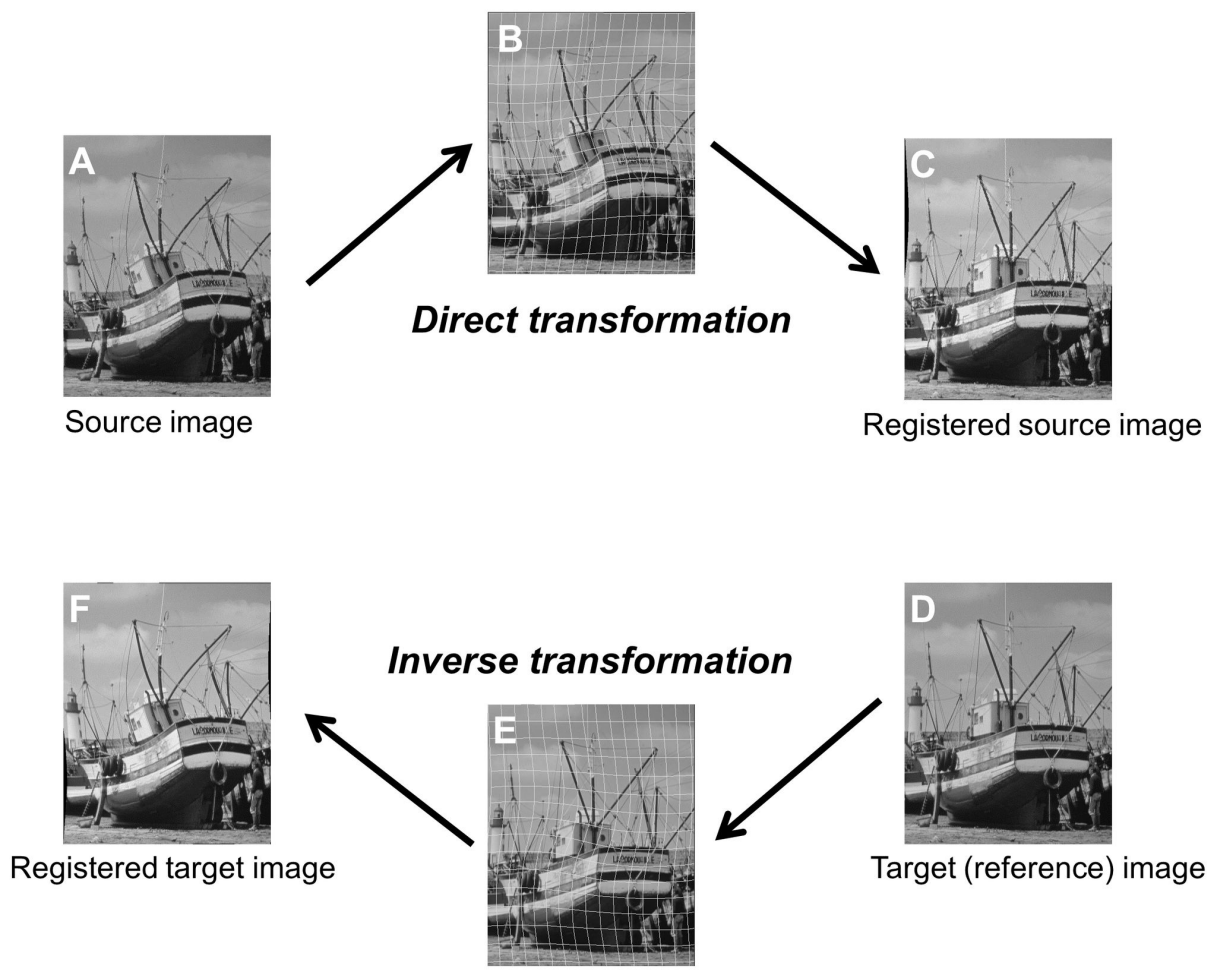
Elastic and Consistent Image Registration. (A) Source image. (B) Deformation grids on (A). Deformation of bUnwarpJ is based on B-spline models. Source image is elastically deformed in order to look as similar as possible to target (reference) image (D). (C) Registered source image. (D) Target (reference) image. (E) Deformation grids on (D). Simultaneously, target image (D) is elastically deformed in order to look as similar as possible to source image (A) to reduce registration error and obtain better correspondence compared to registration without the consistency factor.

After linear image registration, videos were corrected for scanning distortions that were uncorrectable by linear registration and stabilized using the ImageJ plug-in bUnwarpJ, an algorithm for elastic and consistent image registration (Figure 1 and the text of Digital Content S1, which describes the development of the bUnwarpJ algorithm as an ImageJ Plug-in in detail)[32,33]. Deformation of bUnwarpJ was based on B-spline models, and the source image was elastically deformed in order to look as similar as possible to the target (reference) image. Because the bUnwarpJ plug-in was designed for registration between just 2 images and does not perform consecutive processing for registration between the reference image and other remaining frames, bUnwarpJ was run 63 times to analyze each 64-frame (2-s) video. We then created a macro that automates a series of ImageJ commands, and videos were registered automatically (Digital Content S2). Our macro was programmed to use the first frame as a fixed reference frame for image warping by bUnwarpJ.

#### Capillary Visualization

The capillary images were constructed as projections of the moving objects in sequential frames using the motion contrast-enhancement technique reported by Tam et al.[20] Pixels were divided between sequential frames, and the variance of pixels among all division images in each x-y position was calculated to visualize contrast-enhanced capillary images. For each video processed by linear image registration, capillary visualization was performed both before and after elastic image registration, classified as the E(-) and E(+) groups, respectively, for comparison. A macro was also created to facilitate capillary visualization (Digital Content S3).

### Assessment of the Effect of Elastic Registration on Capillary Visualization

The effect of elastic image registration on capillary visualization was assessed by comparing the quality of visualized capillary images between the E(-) and E(+) groups objectively and subjectively.

#### Contrast-to-Noise Ratio Comparison

**Figure 2:**
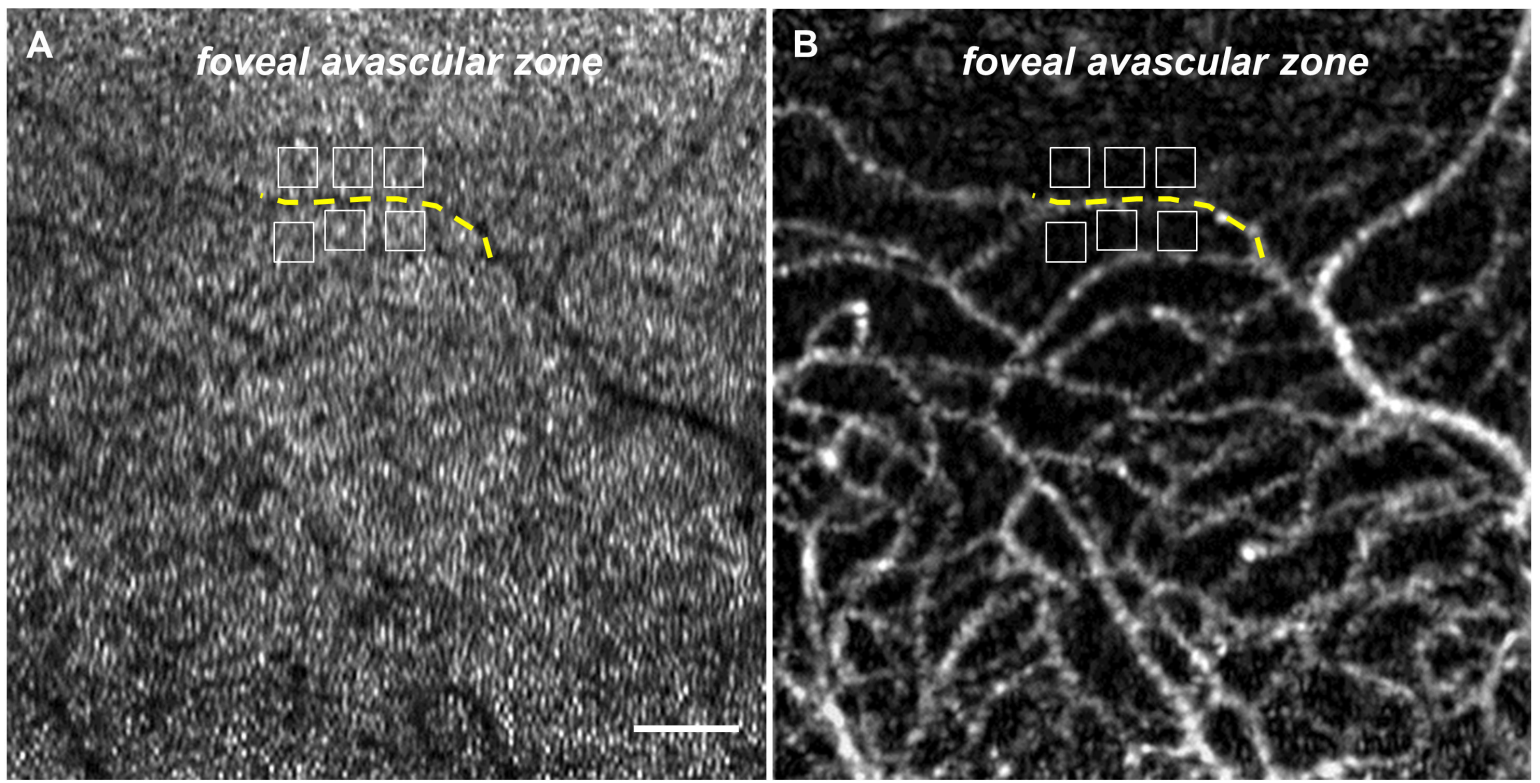
Calculation of Contrast-to-noise Ratio. (A) First frame of video obtained using adaptive optics scanning laser ophthalmoscopy (AO-SLO). Regions of interests (ROI) were set on the first frame of the video. ROIs examined in this study were a line selected on the vessel shadow (yellow dotted line) and areas selected on space surrounding the vessel (white squares) for each subject. Scale bar, 100 μm. (B) Capillary image constructed from the video in (A) after elastic registration. ROIs on (A) were copied to the image with capillary visualization.

To objectively compare image quality, contrast-to-noise ratio (CNR) was calculated for regions of interest (ROI) and compared between pairs of E(-) and E(+)-group images[8,34]. ROIs examined in this study consisted of a line selected on the vessel and areas selected in space surrounding the vessel. To match the position of ROIs between E(-) and E(+) images, we established ROIs on the first frame of the registered videos and copied them to the images with capillary visualization (Figure 2). Briefly, the line was established along a vessel shadow. Six 20 × 20-pixel areas were selected adjacent to the selected capillary shadow on the cone mosaic pattern and then copied to the brightly visualized capillary image using ROI Manager, a built-in function of ImageJ that records the exact location of the ROIs, and the mean gray values of the selected capillary and vessel free space were calculated. CNR was calculated as follows:

$CNR=\left(f-b\right)/\sqrt{\delta_{f}^{2}+\delta_{b}^{2}}$ (1), 

where *f* and *b* are the mean gray values of the ROI set on visualized capillary (foreground) and the 6 ROIs set on vessel free space (background), respectively, and *δ*_*f*_ and *δ*_*b*_ are the standard deviation from the foreground and background mean values, respectively.

#### Expert Comparison

For subjective comparison, 6 experienced ophthalmologists masked to image information performed independent expert comparisons of pairs of E(-) and E(+) images[8,35]. A comparative image quality score for clarity of capillary images was assigned to each image pair as follows: 5 = markedly better capillary image in E(+); 4 = slightly better capillary image in E(+); 3 = equal capillary images in E(+) and E(-); 2 = slightly better capillary image in E(-); and 1 = markedly better capillary image in E(-).

### Measurement of Processing Time Required for Elastic Image Registration

The processing time required for elastic image registration using bUnwarpJ was measured to assess its ease of use and future applicability. Image processing was performed using a Microsoft Windows 7 64-bit operating system with 64-bit central processing unit (Corei7, 2.80 GHz).

### Statistical Analysis

Statistical analysis was performed using StatView version 5.0 (SAS Inc., Cary, NC). All values are presented as the mean ± standard deviation. Paired *t* tests were used to determine CNR differences between the E(-) and E(+) groups. Comparison of the ratio of CNR values in the E(+) and E(-) groups and the mean score assigned by experts between patients and normal subjects was performed using one-way analysis of variance, with post-hoc comparisons tested by the Scheffe procedure. *P* values < 0.05 were considered statistically significant.

## Results

### Effect of Elastic Registration on Capillary Visualization

**Figure 3:**
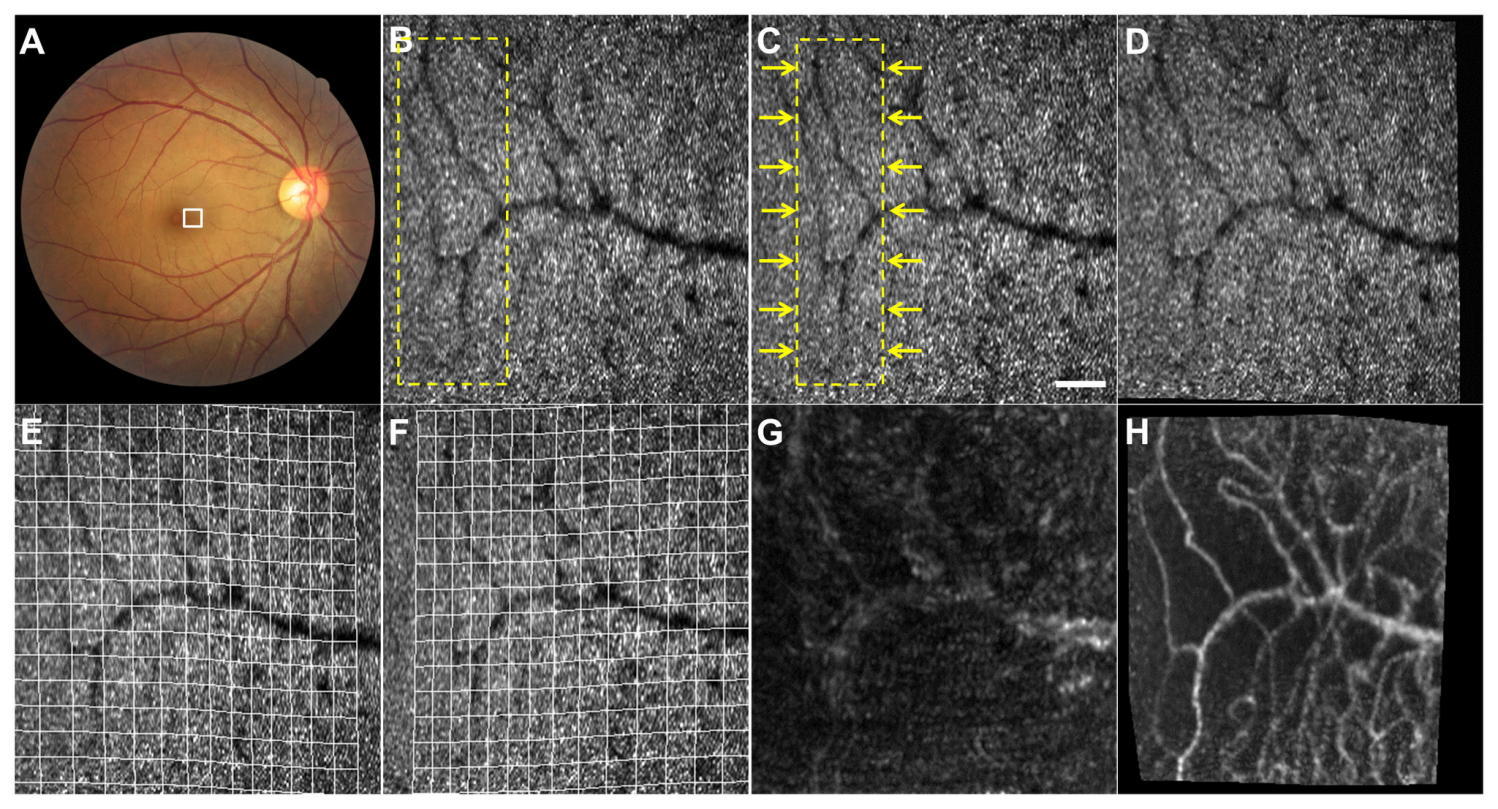
Effect of B-spline-based Elastic Registration on Capillary Visualization. (A) Color fundus photograph of the right eye of a 34-year-old woman with no history of ocular or systemic diseases. (B, C) Two consecutive frames obtained by adaptive optics scanning laser ophthalmoscopy (AO-SLO), which correspond to the area outlined in white in (A) showing scanning distortions that were uncorrectable by linear registration. High-intensity dots representing the cone mosaic pattern and dark shadow of the vessels are observed. Although the squares outlined by a dashed line in (B) and (C) indicate an identical area of the retina, the area in (B) appears to be wider than the area in (C). Scale bar, 100 μm. (D) Registered image of (C), which was elastically deformed to appear as similar as possible to (B). (E, F) Deformation grids on (B) and (C), respectively. (G) Capillary image constructed from unregistered AO-SLO video recorded for 2 s. (H) Capillary image constructed from AO-SLO video after B-spline-based elastic registration. The capillary is more brilliant than that of (G) constructed without registration.

All AO-SLO videos tested in this study were successfully stabilized by elastic image registration with ImageJ, and distortion that was wavy and uncorrectable by linear image registration was considerably diminished (Figure 3 and Digital Content S4). Bright moving objects in the shadows of capillaries on the cone mosaic pattern, considered to be leukocytes or plasma gaps, were easier to observe in stabilized videos than in videos only subjected to linear image registration.

Table 1 shows CNRs for ROIs set on pairs of E(-) and E(+) capillary visualized images in normal subjects and patients with various disease. CNR was significantly higher (*P* < 0.0001) in the E(+) group than the E(-) group (1.92 ± 0.57 vs. 1.24 ± 0.59, respectively) overall. CNR was also significantly higher in the E(+) group than in the E(-) group in normal subjects and each disease group. In no pair of ROIs was the CNR lower in the E(+) group than in the E(-) group. The average ratio of CNR in the E(+) group to CNR in the E(-) group was 2.10 ± 1.73 overall, and no significant difference in this ratio was observed between patients or normal subjects (*P* = 0.379). 

**Table 1:** Differences in Contrast-to-noise Ratio between Images Constructed with or without Elastic Image Registration.

Characteristic	Normal	DR	MacTel	ERM	CSC	Glaucoma	Total
No. eyes	24	5	5	5	5	5	49
Men/women	14/10	3/2	3/2	3/2	5/0	5/0	33/16
Age (y)	35.2 ± 8.1	50.2 ± 7.0	59.4 ± 10.6	65.2 ± 10.6	50.2 ± 12.7	56.6 ± 20.6	46.2 ± 15.4
Processing time (s)	614 ± 198	667 ± 41	530 ± 240	794 ± 211	654 ± 180	544 ± 106	626 ± 190
CNR (E-)	1.30 ± 0.57	1.86 ± 0.37	0.90 ± 0.48	1.02 ± 0.55	1.28 ± 0.62	0.84 ± 0.64	1.24 ± 0.59
CNR (E+)	1.98 ± 0.56	2.18 ± 0.44	1.90 ± 0.52	1.66 ± 0.50	1.98 ± 0.93	1.53 ± 0.31	1.92 ± 0.57
*P* value	< 0.0001	0.0331	0.0113	0.0279	0.0428	0.0164	<0.0001
CNR(E+)/CNR(E-)	1.95 ± 1.55	1.17 ± 0.14	2.93 ± 1.96	2.36 ± 2.07	1.57 ± 0.35	3.21 ± 3.16	2.10 ± 1.73
Expert score	4.21 ± 0.69	3.92 ± 0.58	3.88 ± 0.87	3.40 ± 0.59	3.72 ± 0.42	3.96 ± 0.79	3.99 ± 0.69

DR = diabetic retinopathy; MacTel = idiopathic macular telangiectasia; ERM = epiretinal membrane; CSC = central serous chorioretinopathy; CNR (E-) = contrast-to-noise ratio of images constructed without elastic registration; CNR (E+) = contrast-to-noise ratio of images constructed with elastic registration.

 The mean score assigned by experts was 3.99 ± 0.69 overall, 4.21 ± 0.69 in normal subjects, 3.92 ± 0.58 in DR patients, 3.88 ± 0.87 in MacTel patients, 3.40 ± 0.59 in ERM patients, 3.72 ± 0.42 in CSC patients, and 3.96 ± 0.79 in glaucoma patients, and the mean score did not significantly differ between patients and normal subjects (*P* = 0.306) (Table 1). 

### Processing Time Required for Elastic Image Registration

Mean processing time required for elastic image registration was 626 ± 190 s in total, with no significant difference between patients and normal subjects (*P* = 0.258). Mean processing time was correlated with the area (R = 0.385; *P* = 0.006) and width (R = 0.399; *P* = 0.004) of cropped videos, but not their vertical length (R = 0.169; *P* = 0.248).

### Case 1

**Figure 4:**
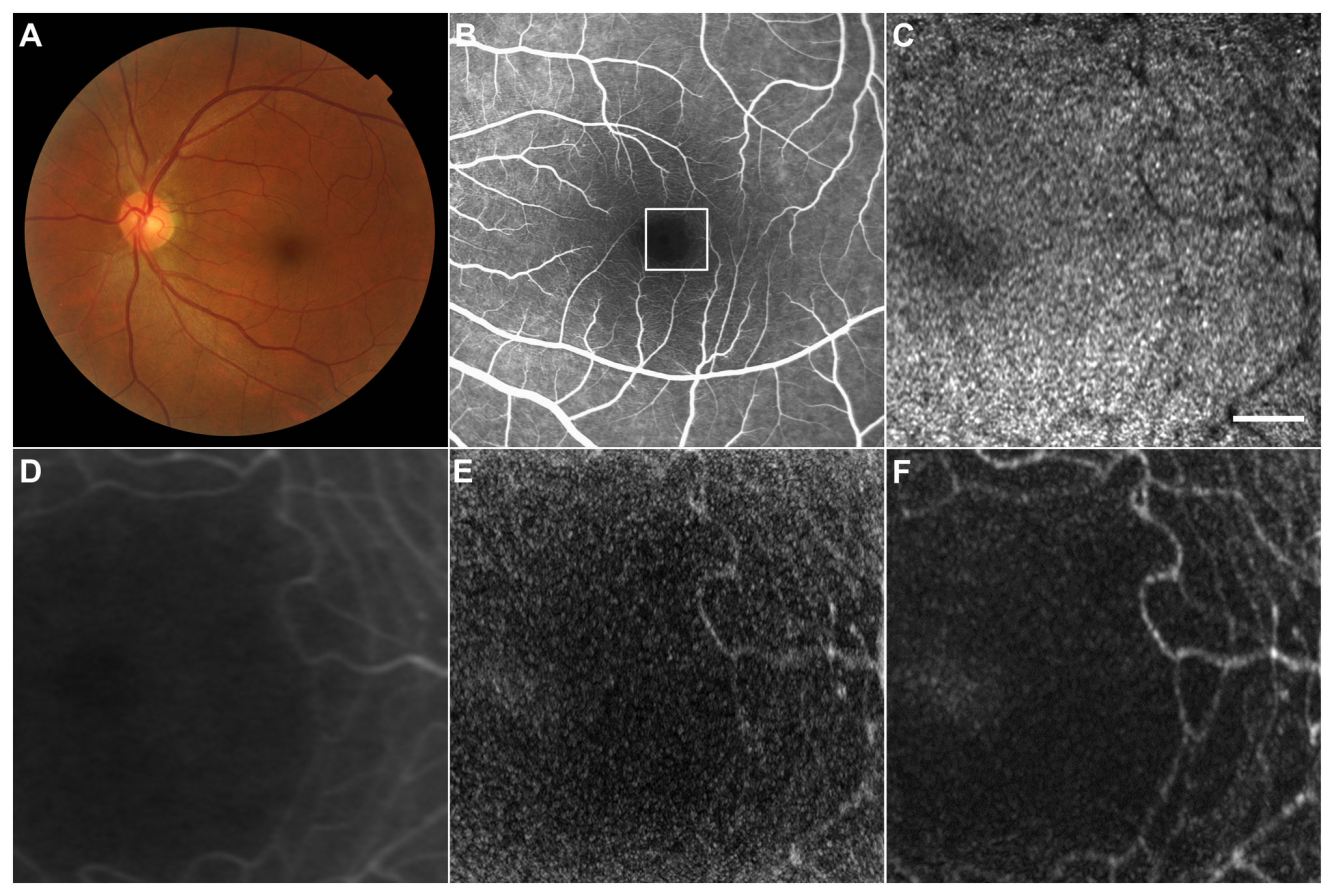
Imaging Results from Case 1. (A) Color fundus photograph. (B) Early-phase fluorescein angiography (FA) image. (C) First frame of the adaptive optics scanning laser ophthalmoscopy (AO-SLO) video, which corresponds to the area outlined in white in (B). (D) FA image. Magnified view of the area outlined in white in (B). (E) Capillary image constructed from unregistered AO-SLO video. (F) Capillary image constructed from AO-SLO video after B-spline-based elastic registration. Scale bar, 100 μm. Note that the capillary image is brilliantly visualized and comparable to early-phase FA image, while the capillary image constructed without elastic registration (E) is rather noisy and blurred.

The parafoveal area of the left eye of a 53-year-old man with no history of ocular or systemic diseases was examined by AO-SLO, and capillary image was constructed from video recorded for 2 s (Figure 4). Capillaries were brilliantly visualized using elastic image registration (Figure 4F), comparably to early-phase fluorescein angiography (FA) images obtained using Heidelberg Retinal Angiography 2 (Heidelberg Engineering) (Figure 4D), while capillary images constructed without elastic registration were rather noisy and blurred (Figure 4E). 

### Case 2

**Figure 5:**
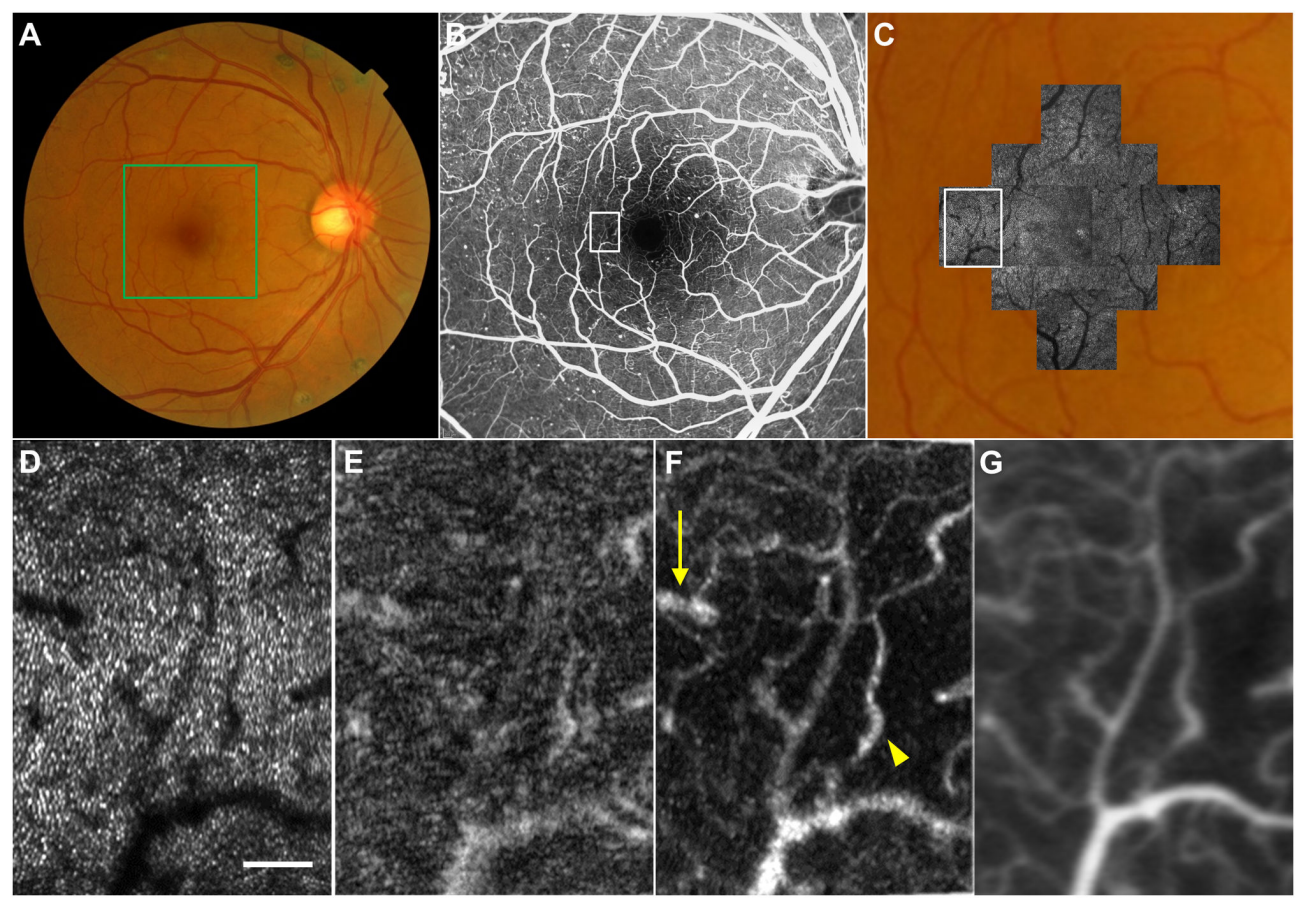
Imaging Results from Case 2. (A) Color fundus photograph. (B) Early-phase fluorescein angiography (FA) image. Microaneurysms are seen as hyperfluorescent dots. (C) Montage of adaptive optics scanning laser ophthalmoscopy (AO-SLO) images overlaid on magnified color fundus photograph corresponding to the area outlined in green in (A). (D) First frame of the AO-SLO video, which corresponds to the area outlined in white in (B) and (C). Scale bar, 100 μm. (E) Capillary image constructed from unregistered AO-SLO video recorded for 2 s. (F) Capillary image constructed from AO-SLO video after B-spline-based elastic registration. Microaneurysm (yellow arrow) as well as caliber variation (yellow arrowhead) are clearly depicted. (G) FA image. Magnified view of the area outlined in white in (B).

A 44-year-old woman with proliferative diabetic retinopathy was referred to us. Panretinal photocoagulation was performed 10 y previously. Her visual acuity was 20/20 in her right eye and 20/20 in her left eye. Parafoveal area of the right eye was scanned by AO-SLO, and capillary image was constructed with and without elastic image registration (Figure 5). In the registered capillary image, microaneurysm as well as caliber variation was depicted clearly in the registered capillary image, and the same findings were also detected in FA.

### Case 3

**Figure 6:**
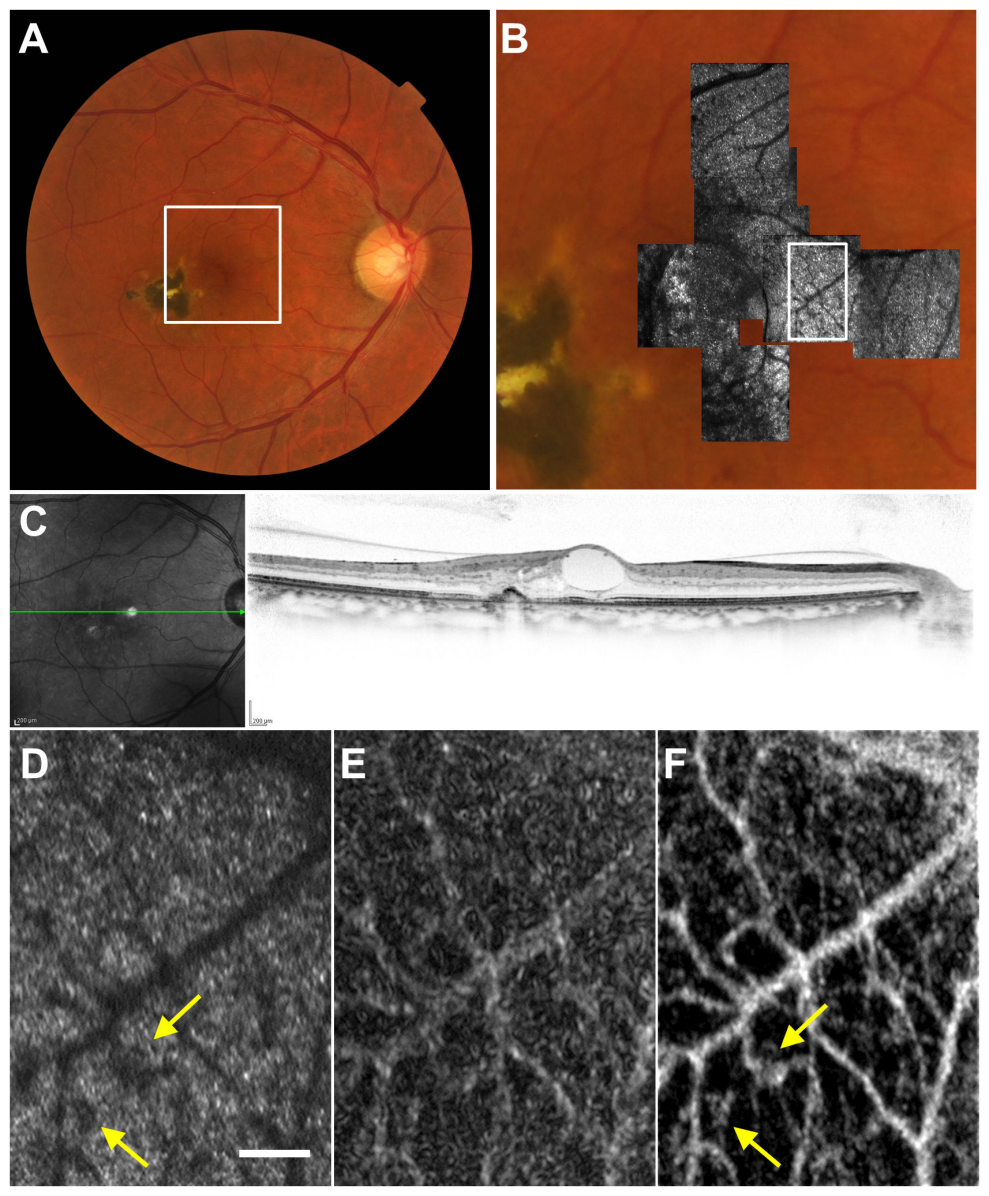
Imaging Results from Case 3. (A) Color fundus photograph. (B) Montage of adaptive optics scanning laser ophthalmoscopy (AO-SLO) images overlaid on magnified color fundus photograph corresponding to the area outlined in white in (A). (C) The optical coherence tomographic scan shows intraretinal cystoid spaces. (D) First frame of the AO-SLO video, which corresponds to the area outlined in white in (B). Dark regions that may correspond to the shadow of microaneurysm on the cone mosaic could be seen along the vessel shadow (arrows). Scale bar, 100 μm. (E) Capillary image constructed from unregistered AO-SLO video. (F) Capillary image constructed from AO-SLO video after B-spline-based elastic registration showing microaneurysm (arrows). The capillary was visualized more clearly and sharply in the image constructed with elastic registration than in the image without elastic registration.

A 46-year-old man with a 6-year history of mild blurring of vision in the right eye was diagnosed with MacTel type 1 (Figure 6). Visual acuity was 20/20 in his right eye. Photocoagulation scar was observed temporal to the fovea. The OCT scan showed intraretinal cystoid spaces[36,37]. AO-SLO video was successfully registered, and the constructed capillary image showed microaneurysm. Capillary was visualized more clearly and sharply in images constructed with elastic registration than in images without elastic registration.

### Case 4

**Figure 7:**
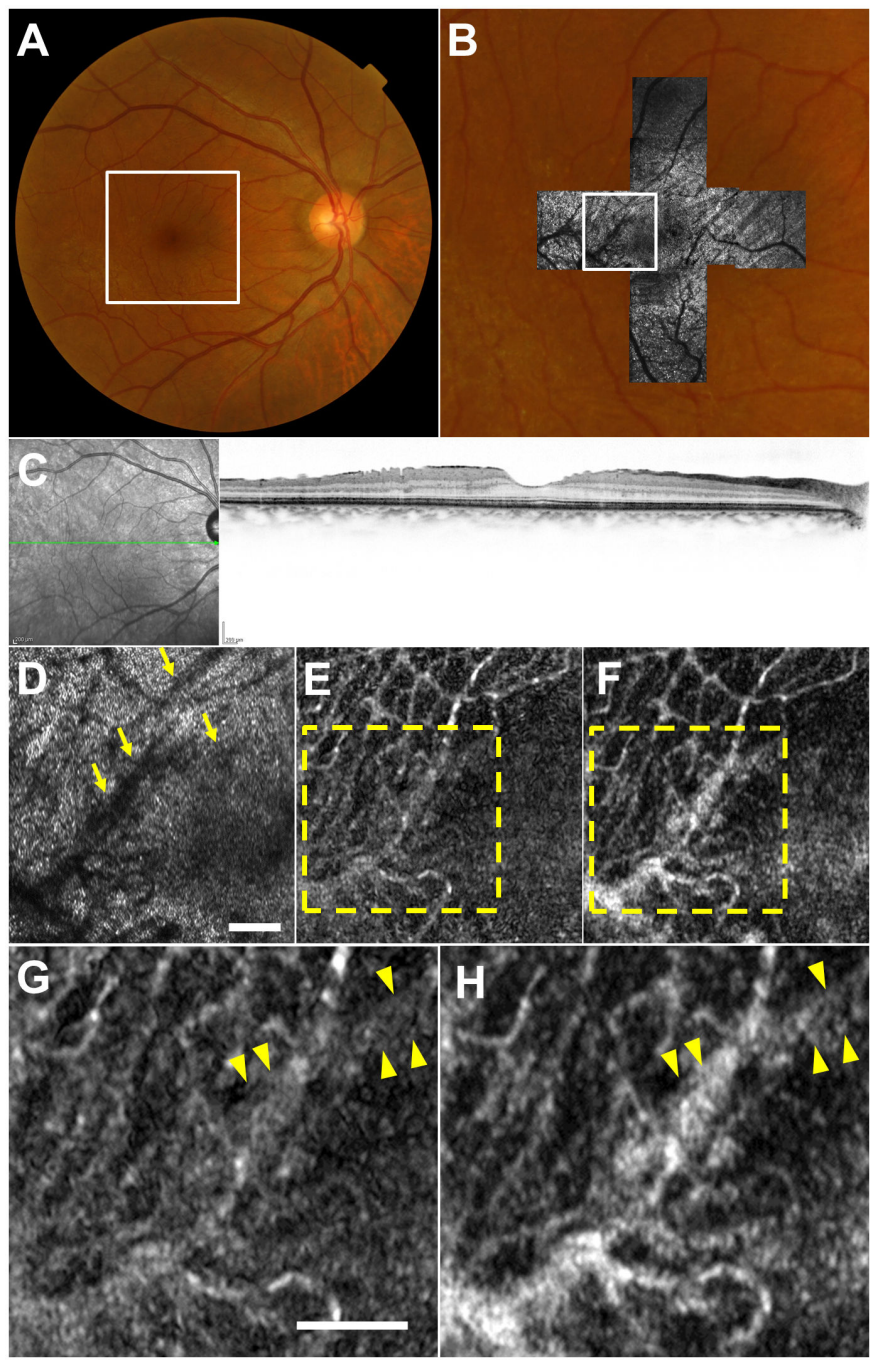
Imaging Results from Case 4. (A) Color fundus photograph. (B) Montage of adaptive optics scanning laser ophthalmoscopy (AO-SLO) images overlaid on magnified color fundus photograph corresponding to the area outlined in white in (A). (C) The optical coherence tomographic scan shows thin epiretinal membrane and retinal folds. (D) First frame of the AO-SLO video, which corresponds to the area outlined in white in (B), shows "microfolds" (arrows). Scale bar, 100 μm. (E) Capillary image constructed from unregistered AO-SLO video. (F) Capillary image constructed from AO-SLO video after B-spline-based elastic registration. (G, H) Magnified view of the area outlined in yellow in (E) and (F). Scale bar, 100 μm. The capillary image was rather noisy in (G), and visible more clearly in (H). Note that vessel images overlapping with microfolds were obscured by white striped artifacts (arrowheads) in both images.

A 65-year-old woman with mild ERM had visual acuity of 20/20 in the right eye (Figure 7). AO-SLO image showed "microfolds" (multiple thin, straight, hyporeflective lines in the photoreceptor layer) as previously reported by Ooto et al.[38] The capillary image was rather noisy in the unregistered image and visible more clearly in the registered image. In both registered and unregistered images, vessel images overlapping with microfolds were obscured by white striped artifacts. These artifacts were not detected in images from patients other than ERM patients and were considered unique to ERM. 

### Case 5

**Figure 8:**
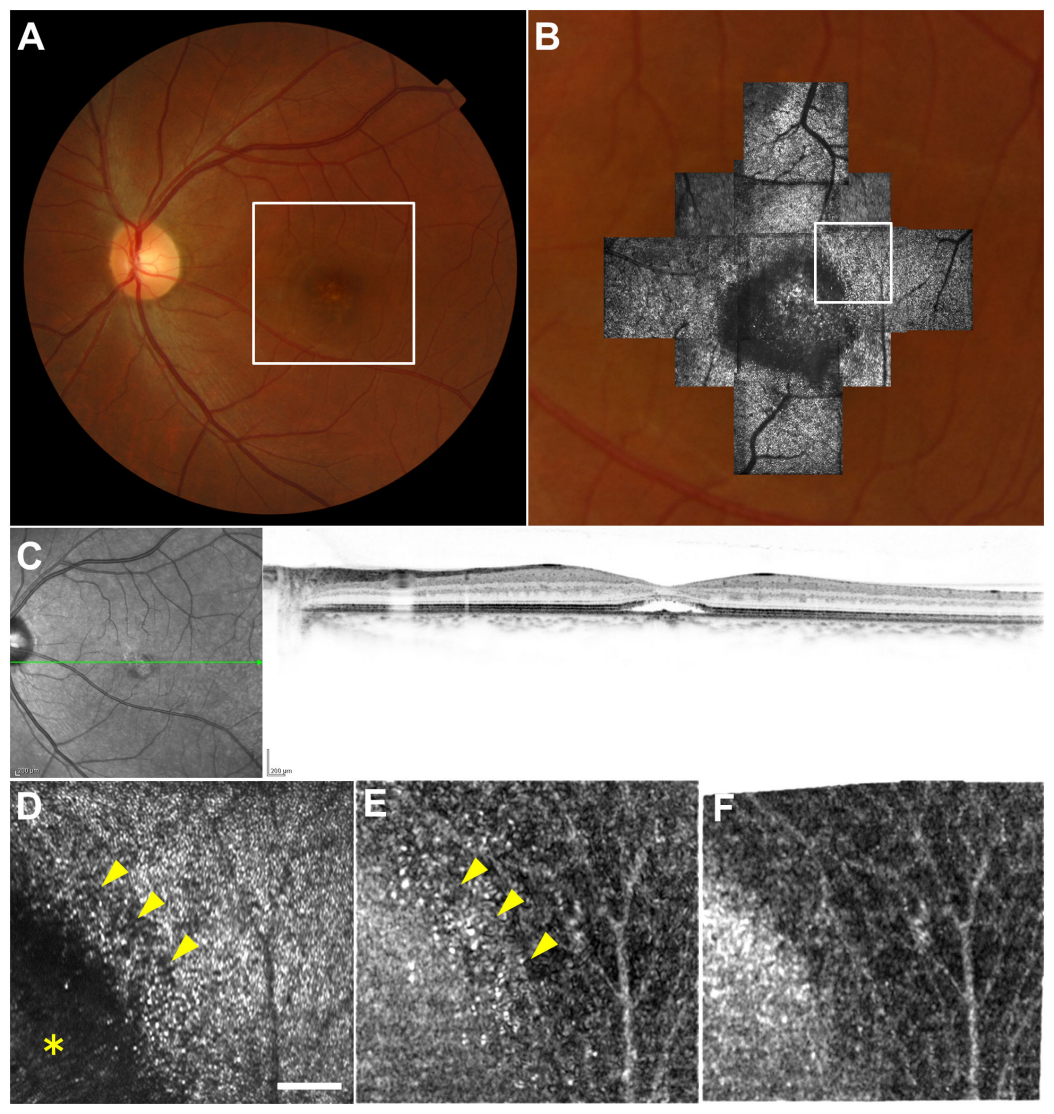
Imaging Results from Case 5. (A) Color fundus photograph. (B) Montage of adaptive optics scanning laser ophthalmoscopy (AO-SLO) images overlaid on magnified color fundus photograph corresponding to the area outlined in white in (A). (C) The optical coherence tomographic scan shows serous retinal detachment and irregularity of the retinal pigment epithelium at the fovea. (D) First frame of the AO-SLO video, which corresponds to the area outlined in white in (B). A dark area is noted on the fovea (asterisk), and many dark patches (arrowheads) representing lost or damaged cones are detected around the dark area. Vessel shadows near the fovea assimilated into the dark area and were not visible. Scale bar, 100 μm. (E) Capillary image constructed from unregistered AO-SLO video. A spotty area was seen in the region containing dark patches (arrowheads). These spots may be due to imperfect alignment. (F) Capillary image constructed from AO-SLO video after B-spline-based elastic registration. The spotty area seen in (E) is not detected.

CSC was diagnosed in the left eye of a 42-year-old man. Visual acuity was 20/50 in the left eye (Figure 8). OCT showed serous retinal detachment and irregularity of the retinal pigment epithelium at the fovea. AO-SLO image of the fovea showed a dark area and many dark patches representing lost or damaged cones, and the pattern of remaining cones was irregular[39]. Vessel shadows near the fovea assimilated into the dark area and were not visible. Capillary images constructed from the video were clearer in the registered image than in the unregistered image, especially in the area with dark patches. A spotty area was seen in the region containing dark patches, and these spots might be due to imperfect alignment.

## Discussion

In this study, we applied B-spline-based elastic registration to AO-SLO videos and demonstrated that the registration enhanced the quality of capillary images constructed from AO-SLO video both subjectively and objectively in normal eyes and eyes with various retinal diseases. CNRs for ROIs set on capillary visualized images were approximately twice as high in the E(+) group as in the E(-) group, and the mean score assigned by experts was 3.97 ± 0.70, suggesting that the registration played an important role in capillary visualization. Visualization of retinal capillaries using AO-SLO was very effective in analyzing microstructures such as microaneurysm and neovascularization, and it may be useful for early detection in patients with diabetes and other retinal vascular disorders[21,22]. The technique requires no contrast dyes, and angiograms can be obtained from the patient without adverse effects. Therefore, it enables frequent acquisition of capillary images from the same patient and may be useful in the evaluation of ophthalmologic or medical treatments in the future.

Capillaries were visualized using motion contrast enhancement as previously reported[20]. Because the technique is based on calculating sequential division images, which are obtained by dividing the gray values of pixels between successive frames, precise registration is required to cancel the image of the mosaic pattern outside of the vessel shadow and extract only the motion of blood cells. In our protocol for capillary visualization, comparatively short (2-s) recording time and wide recording field (2.8 × 2.8°) were adopted to minimize the inter-frame position gap between scan frames produced by eye motion. Although the protocol was also convenient for simultaneous acquisition of the wide-field capillary network, inadequate registration would fail to cancel the background mosaic pattern by pixel and result in spotty areas on the image as in the example shown in Figure 8. 

 B-spline-based deformation models are control grids composed of B-spline curves, which are smooth curves defined by the given control points[32,33]. Using the deformation models, pixels in an image from AO-SLO video can be matched to pixels on the reference image to obtain interpolated images warped by a spline function. B-spline-based elastic image registrations have been utilized widely in many biomedical imaging problems and have proven suitable for the type of deformations encountered with magnetic resonance imaging[23,24,26,27,40]. Other than the registration method applied here, several approaches have been reported to date. Arathorn et al. reported cross-correlation methods, whereby the frames were cut in rectangles and displaced in the x and y directions to correlate them to a reference frame[17,18]. Since the distortions created by intraframe eye motion are mainly due to the raster pattern of scanning, which is relatively slower than scanning line, breaking up the frames into strips parallel to the fast scanning mirror direction is reasonable. In fact, our results on the processing time required for elastic image registration support the adequacy of cross-correlation methods. The mean processing time was correlated with the width but not vertical length of cropped videos, suggesting that many more distortions are present in the horizontal direction, which is perpendicular to the fast scanning mirror direction in our system, than in the vertical. In the KLT-SIFT algorithm[14], stable point features were extracted from AO-SLO images using the SIFT algorithm[41], and features were tracked from frame to frame using the KLT algorithm, which has low computational complexity and is considered faster than traditional techniques[42], followed by second-order polynomial transformation to remove distortions. Using KLT-SIFT, features on 30-Hz AO-SLO video can be tracked in real-time. 

Mean processing time required for elastic image registration was approximately 20 s per frame in this study. Compared to the 2 above-mentioned registration methods, which can compute registration on a millisecond time scale per frame, the registration method tested here required substantial computing time. Although our goal is not to track single cones or stabilized stimulus delivery to the living retina in real-time, 10-min processing time to register a 2-s video is considered to be too long for applying the method widely to future investigations of AO-SLO-assisted blood flow analysis. The use of general-purpose software and the consistent image registration that is included in the energy function of the registration software appeared to factor in the long processing time. However, putting aside the issue of processing time, B-spline-based elastic image registration provides high-quality interpolation because of its precise pixel-wise registration and enables capillary visualization constructed from short-term recording. Development of special tools to integrate software, applications, and systems from different vendors may improve availability and speed.

Public domain software ImageJ and associated plug-ins were used for image processing including image registration and capillary visualization in the current study based on their ready availability[31,32]. Anyone wishing to try the registration and capillary visualization described here need only download the software and macro texts. Currently, AO-SLO machines are being developed by researchers in many countries, and more groups will participate in this new area of research in the future. We believe that the information described in this paper will support the quick initiation of research on AO-SLO-assisted blood flow analysis, which will in turn facilitate investigations of retinal microcirculation. Using B-spline-based elastic image registration, capillaries were visualized well without contrast dyes, similar to FA, in all diseased eyes except cases of ERM and CSC. In ERM, vessel images overlapping with “microfolds” were obscured by white cloud-like artifacts[38]. In CSC, vessel shadows near the fovea assimilated into the dark area and were not visible[39]. Ultimately, capillaries in these areas could not be visualized. Because AO-SLO was focused on the photoreceptor layer and blood cells were observed as shadings on the shadows of the bright cone mosaic patterns of photoreceptors, this focusing may be the cause of these artifacts and loss of capillary images[30]. As previously described, the characteristics of the reflected AO-SLO laser from photoreceptors are affected by blood cells because of their different scattering coefficients[43]. When the scanning is focused on the photoreceptor layer, leukocytes and blood plasma are candidates for the bright particles moving in the dark vessel shadows due to the low absorptivity of the AO-SLO laser, and erythrocytes are candidates for the region that is darker than the vessel shadow because they block the AO-SLO lasers. Accordingly, the presence of structures that can block the laser or absences of photoreceptors that can reflect the laser as in the case of microfolds and loss of cone mosaic pattern will lead to the failure of blood flow detection. Although a possible solution to the problem may be focusing the scanning layer on the capillary layer, further investigation is needed to assess the optimal focus for capillary visualization using AO-SLO.

Our study has several limitations: (1) Because the registration was computed after video recording without real time eye-tracking, video recording was limited to a short duration to minimize the inter-frame position gap between scan frames. Longer-duration video recording may fail to construct adequately wide-area capillary images (2). The first frame of the video was used as a fixed reference frame for image warping. Because the reference frame itself can contain substantial distortions produced by intraframe eye movement, subsequent source frames and constructed capillary images will contain the same distortions. To overcome this issue, registration methods may be altered in the future to adopt a method based on analysis of eye movement, which has the potential to select a reference frame with minimum distortion [44].

In conclusion, use of B-spline-based elastic image registration in AO-SLO-assisted capillary visualization was found to be both objectively and subjectively effective for enhancing image quality. Its high-quality interpolation and ready availability may further facilitate the application of AO-SLO for the study of retinal microcirculation.

## Supporting Information

Digital Content S1 **Detailed description of the algorithm of bUnwarpJ Developed as an ImageJ Plug-in.**
(DOCX)

Digital Content S2 **Macro for Continuous Elastic Image Registration Using bUnwarpJ Developed as an ImageJ Plug-in.** Before elastic image registration, videos were cropped to eliminate the margin without retinal image, which was a by-product of registration. Our macro was programmed to use the first frame as a fixed reference frame for image warping by bUnwarpJ. Note that the advanced setting of bUnwarpJ was modified to function successfully in our AO-SLO images.
(TIF)

Digital Content S3 **Macro for Capillary Visualization.** The capillary images were constructed as projections of the moving objects in sequential frames using the motion contrast enhancement with this macro. In accordance with the total frame number of the video, the original data for frame number, indicated as 64 on the second line and the fifth line from the top, need to be rewritten.
(TIF)

Digital Content S4 **Movies with and without Elastic Image Registration.** The video field size is 674 × 705 μm^2^ each, and the frame rate is 32 fps. Distortion that was wavy and uncorrectable by linear image registration was considerably diminished in the video with elastic image registration (right side) compared to video without elastic image registration (Left Side).
(MOV)
