# Supplementary material for: Image Quality Improvement in Adaptive Optics Scanning Laser Ophthalmoscopy Assisted Capillary Visualization Using B-spline-based Elastic Image Registration
Source: PLoS One. 2013 Nov 12;8(11):e80106. doi: 10.1371/journal.pone.0080106 (PMC3827159; doi:10.1371/journal.pone.0080106)
Supplement: Digital Content S1 — Detailed description of the algorithm of bUnwarpJ Developed as an ImageJ Plug-in. (DOCX) [file pone.0080106.s001.docx]

Deformation of bUnwarpJ is based on B-spline models, and registration was performed by minimizing the following energy functional E:

$E=\omega_{i}E_{img}+\omega_{\mu}E_{\mu}+\left(\omega_{d}E_{div}+\omega_{r}E_{rot}\right)+\omega_{c}E_{cons}$ (1),

where E_img_ is the dissimilarity of image gray values between the reference image and warped source image, E_μ_ is the optional landmark constraint, ω_d_ E_div_ + ω_r_ E_rot_ is the regularizer related to gradients of divergence and curl of the deformation vectors, and E_cons_ is the geometrical consistency between the direct transformation (from source to target) and the “inverse” transformation (from target to source) (Figure 1). Using this energy function, registration is performed on a computer to match the distributions of gray values in the 2 images (E_img_) with soft constraints by landmarks (E_μ_) and with robust deformation when little information is available (ω_d_ E_div_ + ω_r_ E_rot_). Simultaneously, this method contains a consistent image registration factor (E_cons_) to reduce registration error and obtain better correspondence compared to registration without the consistency factor in the energy function.
