# Supplementary figures and images for: Image Quality Improvement in Adaptive Optics Scanning Laser Ophthalmoscopy Assisted Capillary Visualization Using B-spline-based Elastic Image Registration
Source: PLoS One. 2013 Nov 12;8(11):e80106. doi: 10.1371/journal.pone.0080106 (PMC3827159; doi:10.1371/journal.pone.0080106)

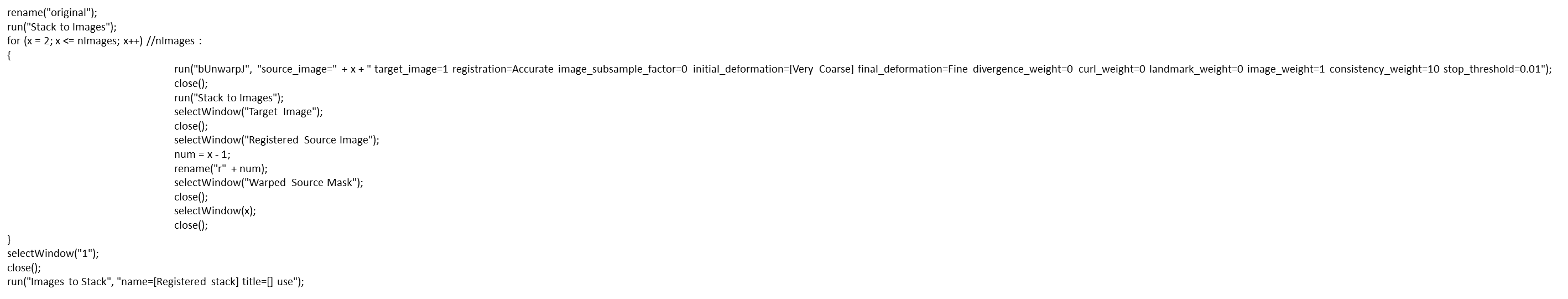

Supplement: Digital Content S2 — Macro for Continuous Elastic Image Registration Using bUnwarpJ Developed as an ImageJ Plug-in. Before elastic image registration, videos were cropped to eliminate the margin without retinal image, which was a by-product of registration. Our macro was programmed to use the first frame as a fixed reference frame for image warping by bUnwarpJ. Note that the advanced setting of bUnwarpJ was modified to function successfully in our AO-SLO images. (TIF) [file pone.0080106.s002.tif]

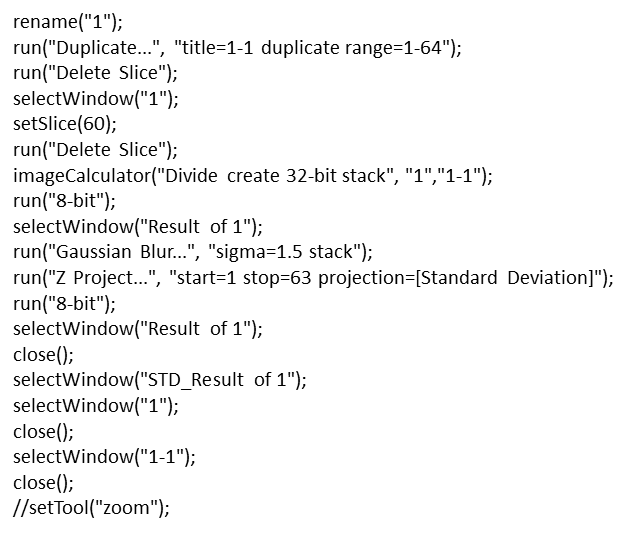

Supplement: Digital Content S3 — Macro for Capillary Visualization. The capillary images were constructed as projections of the moving objects in sequential frames using the motion contrast enhancement with this macro. In accordance with the total frame number of the video, the original data for frame number, indicated as 64 on the second line and the fifth line from the top, need to be rewritten. (TIF) [file pone.0080106.s003.tif]
